# Non-Squamous Cell Carcinoma of the Larynx: A State-of-the-Art Review

**DOI:** 10.3390/jpm13071084

**Published:** 2023-06-30

**Authors:** Carlos M. Chiesa-Estomba, Maria Rosaria Barillari, Miguel Mayo-Yáñez, Antonino Maniaci, Nicolas Fakhry, Giovanni Cammaroto, Tareck Ayad, Jerome R. Lechien

**Affiliations:** 1 Department of Otorhinolaryngology, Donostia University Hospital, Osakidetza, 20014 San Sebastian, Spain; 2 Head & Neck Study Group, Young-Otolaryngologists of the International Federations of Oto-Rhino-Laryngological Societies (YO-IFOS), 13005 Marseille, Francenicolas.fakhry@ap-hm.fr (N.F.);; 3 Division of Phoniatrics and Audiology, Department of Mental and Physical Health and Preventive Medicine, University of L. Vanvitelli, 81100 Naples, Italy; 4 Otorhinolaryngology—Head and Neck Surgery Department, Complexo Hospitalario Universitario A Coruña (CHUAC), 15006 A Coruña, Spain; 5 Department of Medical and Surgical Sciences and Advanced Technologies “GF Ingrassia”, ENT Section, University of Catania, Via S. Sofia 78, 95125 Catania, Italy; 6 Department of Otorhinolaryngology-Head and Neck Surgery, APHM, La Conception University Hospital, 13005 Marseille, France; 7 Department of Otolaryngology—Head & Neck Surgery, Morgagni Pierantoni Hospital, 47100 Forli, Italy; 8 Division of Otolaryngology—Head & Neck Surgery, Centre Hospitalier de L’Université de Montréal, Montreal, QC H2X 0C1, Canada; 9 Department of Otolaryngology—Head & Neck Surgery, Foch Hospital, School of Medicine, UFR Simone Veil, Université Versailles Saint-Quentin-en-Yvelines (Paris Saclay University), 91190 Paris, France

**Keywords:** non-squamous, laryngeal, carcinoma, sarcoma

## Abstract

(1) Background: Non-squamous cell laryngeal carcinoma includes endothelial tumors, such as minor salivary gland tumors, lymphoepithelial tumors, neuroendocrine tumors, soft and hard tissue sarcomas, and malignant melanomas. (2) Methods: A state-of-the-art review using the MEDLINE/PUBMED, Google Scholar, Ovid Medline, Embase, and Scopus electronic databases was performed. (3) Conclusions: In order to optimize overall treatment outcomes, a multidisciplinary, patient-centered approach to the management of non-SCC of the larynx must be adopted universally; a national or international registry on non-SCC laryngeal cancer can be useful to improve understanding about the behavior of this kind of tumor.

## 1. Introduction

Laryngeal carcinomas represent one third of all head and neck cancers [1], with an incidence rate of 5% [2] and an estimated account of 13,000 newly diagnosed cases each year in the USA [3]. Of these, it is estimated that 95% of all laryngeal cancers (LC) are squamous cell carcinomas (SCC) [4], while the remaining cases fall under a group defined as “non-squamous cell carcinoma” (non-SCC). Non-SCC LC includes endothelial tumors, such as minor salivary gland tumors, lymphoepithelial tumors, neuroendocrine tumors, soft and hard tissue sarcomas, and malignant melanomas [5].

Despite the increasing knowledge about the treatment of LC in the last decades, the lack of information about non-SCC LC makes it difficult to establish a proper patient profile, define outcomes, or improve patient counseling. Additionally, the literature is scarce regarding these types of tumors [4,5,6,7,8,9]. The current guidelines from the National Comprehensive Cancer Network make no direct recommendation for the treatment of non-SCC LC [10] but, depending on the disease’s extent and histology, a multidisciplinary approach (surgery, radiotherapy, or systemic therapy) can be recommended.

The objective of this review was to systematically evaluate the available literature and current knowledge about primary non-SCC LC, identify future research lines on this topic, and explore potential personalized approaches.

## 2. Materials and Methods

This state-of-the-art review involved the MEDLINE/PUBMED, Google Scholar, Ovid Medline, Embase, and Scopus electronic databases, with search terms: “non-squamous laryngeal cancer”, “non-squamous glottic cancer”, “non-squamous head and neck cancer”, “non-squamous laryngeal cancer treatment”, “laryngeal sarcoma”, “neuroendocrine laryngeal carcinoma”, “laryngeal salivary gland carcinoma”, “laryngeal lymphoma”, “laryngeal melanoma”, and “laryngeal spindle-cell carcinoma”. Clinical prospective and retrospective studies, experimental research, meta-analyses, and systematic reviews published on non-SCC LC until October 2022 were included in the study, while case reports, publications focusing on non-SCC in children, and grey literature were excluded. Articles were required to be written in English, Italian, French, or Spanish. Despite not being a systematic review, article selection was based on the recommendations of the Preferred Reporting Items for Systematic reviews and Meta-Analyses (PRISMA) criteria [11]. Titles and abstracts were screened by two investigators (C.M.C.E. and M.R.B.) to discard irrelevant or duplicate publications. Once the duplicates were refined and the articles selected, the authors reviewed the complete texts of all the manuscripts and the bibliographic references with the aim of including possible studies not found through the search strategy.

A critical analysis of the literature was then performed focusing on histology, incidence and prevalence, clinical presentation, diagnosis, and treatment. Ethics committee approval was not required for this review.

## 3. Discussion

Non-SCC of the larynx represents a unique diagnostic and therapeutic challenge for clinicians because these lesions are encountered infrequently. According to the available evidence, almost all benign and malignant laryngeal neoplasms are epithelial in origin, and all of them arise from the squamous epithelium which covers the larynx. Other non-SCC LC that are usually considered separately, despite their epithelial origin, correspond to spindle-cell carcinoma or salivary gland tumors (Table 1).

These neoplasms usually originate from the submucosal region and the fibrocartilaginous skeleton (cartilage, nerve trunks, minor salivary glands, blood, and lymphatic vessels) and they appear, objectively, as laryngeal swellings covered by normal mucosa. According to the existing literature, the majority of patients affected by non-SCC carcinomas were aged between 55 and 75 years; male subjects are considered at higher risk for non-SCCs compared to females (1.7:1 ratio) [12,13,14], except for lymphoepithelial carcinoma, which is more common in women [12]. In terms of incidence, the most prevalent laryngeal non-SCCs are neuroendocrine tumors (37%) [13,14,15].

### 3.1. Diagnostic Workup

As these tumors are rare, there are limited data regarding optimal treatment modality based on histologic type. Because survival and staging data are limited, there is currently no consensus on management principles for most of these neoplasms.

Clinical symptoms are similar in patients affected by SCCs and in those affected by non-SCC, and they are mainly represented by hoarseness followed by dyspnea/stridor and dysphagia. Tobacco smoking and alcohol abuse, contrary to SCC, do not seem to be considered predominant risk factors for non-SCCs [16,17].

The routine clinical approach includes visualization of the larynx with flexible laryngoscopy and subsequent biopsy (in-office procedure or through direct laryngoscopy) at directed sites of suspicion. SCC may be recognized during flexible laryngoscopy as evident mucosal abnormalities, whereas non-SCC laryngeal tumors usually show up as submucosal masses, limiting the possibilities of this diagnostic approach alone. So, especially in case of persistence/worsening of symptoms, a radiographic evaluation is mandatory to confirm the diagnosis [18].

The first choice usually corresponds to computed tomography (CT), because this provides a high anatomical resolution, allowing us to determine the exact location, vascularization, and extension of the lesion, as well as the involvement of the laryngeal skeleton [19]. The second choice usually correspond to magnetic resonance imaging (MRI), which may provide a more accurate and detailed description of laryngeal subsites, anterior commissure, submucosal infiltration into pre- and paraglottic spaces, cartilage, and base of tongue invasion, as well as metastasis to regional lymph nodes in the neck [20]. Histological examination usually needs to be supported by a comprehensive immunohistochemical examination.

### 3.2. Neuroendocrine Tumors

Neuroendocrine laryngeal malignancies (NLM) are uncommon and heterogeneous tumors, which share some specific morphological, histochemical, immunohistochemical, and ultrastructural characteristics but may present different prognosis on the basis of the tumor type [21]. According to a recent population-based analysis published by Torabi et al., NLM represent 37% of non-SCC of the larynx [12]. Moreover, patients affected by neuroendocrine tumors have been recognized as those with the highest rate of metastases, and with the lowest survival among all histological subtypes [21].

NLM are divided into two broad categories based on their tissue of origin: epithelial and neural. the most common subtypes usually correspond to typical carcinoid, atypical carcinoid, and small-cell undifferentiated carcinoma. In 2017, a new classification developed by the WHO modified the terminology and classification of laryngeal neuroendocrine carcinomas into well-, moderately, and poorly differentiated neuroendocrine carcinoma. Moreover, this classification emphasizes the relevance of poorly differentiated neuroendocrine carcinomas including the poorly differentiated neuroendocrine carcinomas of a small cell type (SCNEC) and a large cell type (LCNEC) [22].

The diagnosis of neuroendocrine neoplasm requires the presence of both neuroendocrine morphologies via light microscopy and the demonstration of neuroendocrine differentiation by means of immunohistochemistry or electron microscopy. NLM can appear as an infiltrative laryngeal mass on imaging, but the findings are nonspecific and can resemble those of SCC. Furthermore, there may be accompanying bulky metastatic cervical lymphadenopathy, typically without necrosis or cystic changes [23]. Primary SCNEC of the larynx may be associated with paraneoplastic syndromes, akin to small-cell cancer of the lung [24].

Regarding treatment options, due to the recent change in terminology, the literature available is scarce. However, based on previous data, carcinoid tumors (typical and atypical) are usually treated by surgery (partial or total laryngectomy) [25,26,27], while neck management can be different. In particular, in patients affected by typical carcinoid, neck dissection is not mandatory due to the low incidence of neck metastasis [14]; on the other hand, in the atypical carcinoid, elective or therapeutic neck dissection is necessary including at least levels IIa and III [14,28]. By contrast, the best option to achieve local control of SCNEC laryngeal tumors continues to be concurrent chemoradiation therapy [29].

As proposed in 1986 by Baugh et al., radiation alone does not improve survival, but it may increase local control. Adjuvant chemotherapy increases the median survival from 11 to 19 months [30], and the concomitant CRT treatment resulted in a median survival of 55 months. However, chemotherapy resistance has been described as an important indicator of poor prognosis [15].

Regarding SCNEC, we need to consider some key concepts. Firstly, the most common location affected in the H&N region is the larynx, representing at least 35% of patients [31]. Secondly, local control is often insufficient to prevent distant metastasis, and nearly 90% of these patients may develop distant recurrence that leads to death [15,29].

Another relevant factor that is important to keep in mind corresponds to the differential diagnosis among primary laryngeal neuroendocrine malignancies and laryngeal metastasis from a primary small-cell neuroendocrine carcinoma of the lung, where imaging studies of the lung want to be mandatory in order to obtain a more precise differential diagnosis [32]. Despite laryngeal neuroendocrine metastasis being extremely rare, two case reports have been described related to a prostate [33] and aggressive rectal carcinoid metastasizing to the larynx [34].

### 3.3. Spindle-Cell Carcinoma

Spindle-cell carcinoma (SPCC) is composed of spindle-cell elements. Histologically, the spindle-cell elements are derived from squamous epithelium but they follow a divergent mesenchymal differentiation, and usually can be misdiagnosed due to their histological appearance [35]. These tumors usually arise in the glottis, looking like a polypoid or a pedicled polyp mass [36,37]. Surgery is considered the first-line treatment, a 5-year OS rate range between 54 and 59% is reported [36,37]. The largest series to date [38] challenges the established view of SPCC as an aggressive variant of SCC. Although typically a higher grade at diagnosis, SPCC has less frequent nodal involvement than SCC; SPCC and SCC have similar incidence of distant metastasis; and SPCC is not associated with a higher hazard ratio of death, suggesting that SPCC histology is not an independent adverse prognostic factor, which should be considered when counseling patients with SPCC [38].

### 3.4. Minor Salivary Gland Tumors

Malignant salivary gland carcinoma (MSGC) of the larynx includes different etiologies like: adenosquamous carcinoma (ASC), mucoepidermoid carcinoma (MEC), adenoid cystic carcinoma (ACC), and adenocarcinoma. The etiology of these tumors remains unknown, and tumor distribution wants to be correlated with the anatomical distribution of submucosal minor salivary glands in the larynx, which are mainly located in the region of the false vocal cords, ventricles, and the anterior subglottic commissure [39].

These tumors tend to be indolent and spread submucosally as a mass with an intact overlying mucosal surface, which may result in delayed diagnosis. Moreover, there are some characteristics that are specific for each histological subtype.

**Figure 1:**
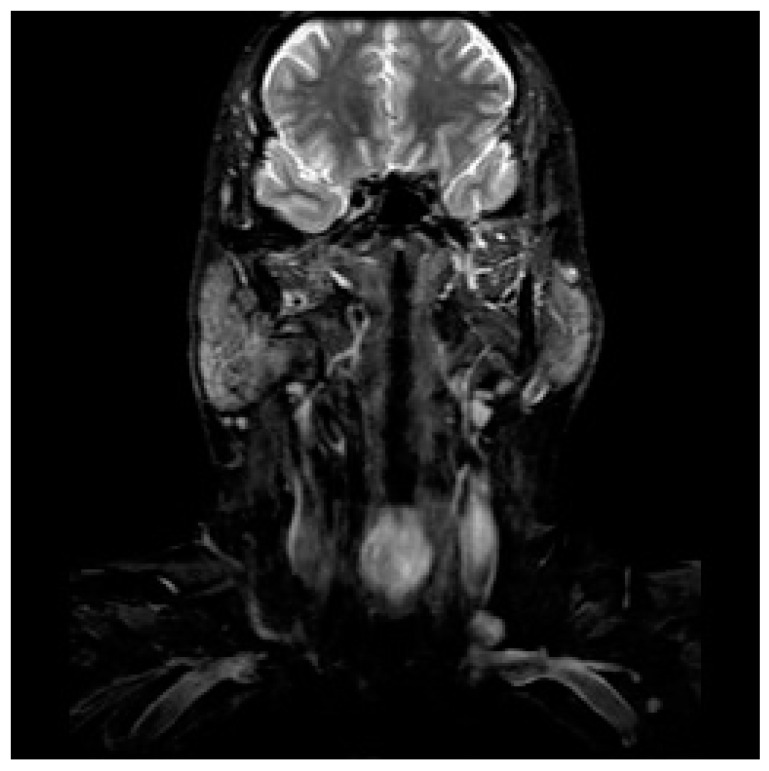
MRI scan of a patients with a laryngeal adenoid cystic carcinoma.

ACC is the most common type of laryngeal MSGC, representing <1% of all tumors affecting the larynx [40]. Despite there not being a clear predominance by sex, these tumors are more common in the fifth decade of life and are not correlated with tobacco consumption. ACC patients are rarely affected by cervical lymph node metastasis and are characterized by multiple recurrences as late distant metastasis [41]. By contrast, ASC is more common in men aged 60–70 years, and has a high propensity for metastasis [41,42] (Figure 1).

Mucoepidermoid carcinoma (MEC) represents unusual histology in the larynx, predominantly affecting men aged 50–60 years [43]. It is thought that this tumor arises from the excretory ducts of seromucous glands and has a wide spectrum of clinical behavior from locally invasive to highly malignant [44], often corresponding to tumor grade and can be divided into high differentiation type and low differentiation type, according to the proportion of different cells in the tumor and the degree of differentiation [45]. Regarding tumor localization, 60% of tumors affect the supraglottic space, 30% the glottis, and 10% the subglottic or want to be a trans-glottic lesion [46,47]. Historically, patients affected by MEC were treated by means of total laryngectomy, while partial laryngectomy is considered suboptimal due to the submucosal spread of these lesions [47,48,49], with the exception of small, localized, and low-grade tumors [48]. Neck dissection is indicated in high-grade tumors and in patients with positive lymph nodes [50,51], and postoperative RT needs to be considered in patients affected by medium- and high-grade MEC, positive margins, and extracapsular spread. RT alone has been described in early-stage/low-grade cases [52]. However, MEC are considered moderately radiosensitive tumors, and for this reason, RT exclusively is not taken into consideration as a first option [53]. Five-year overall survival rates reported in the literature range from 0 to 45% in high-grade MEC, from 60 to 90% in intermediate-grade MEC, and from 90 to 100% in low-grade MEC [54].

Despite data concerning treatment options and outcomes are scarce, surgery is still considered the treatment of choice. The potential role of adjuvant RT has not been well defined yet, although there is an agreement that it should be considered complementary in advanced-stage or high-grade disease [40].

### 3.5. Soft Tissue Sarcomas

**Figure 2:**
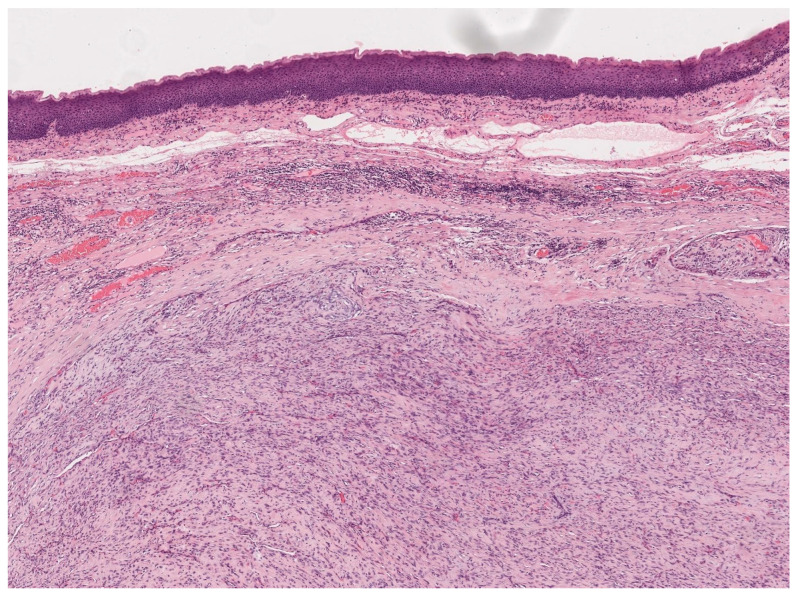
Histological images (hematoxylin–eosin) of a patient with a laryngeal well-differentiated liposarcoma.

**Figure 3:**
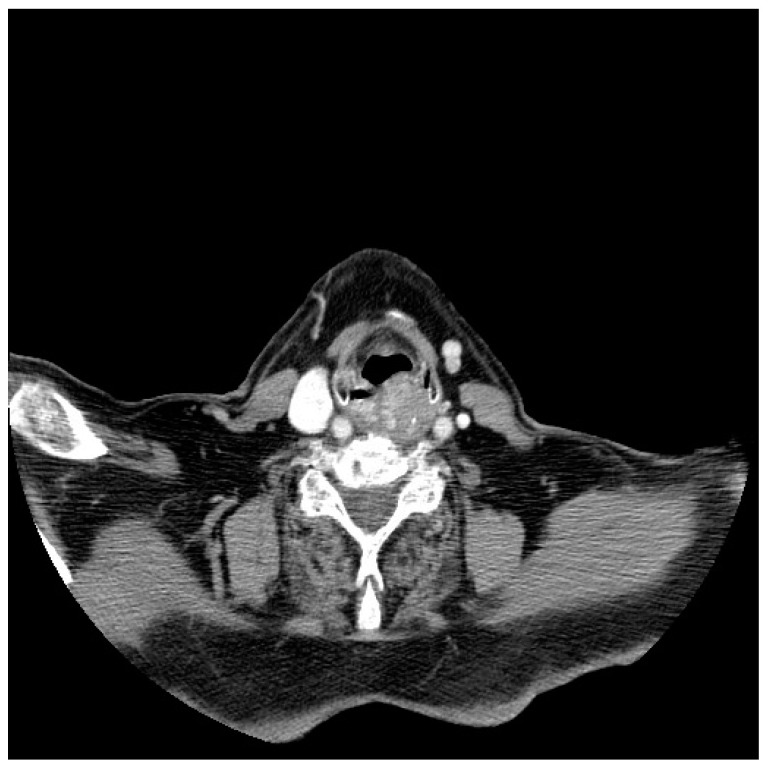
CT scan of a patient with a laryngeal well-differentiated liposarcoma.

They represent less than 1% of all laryngeal tumors. A variety of uncommon sarcoma subtypes arising from the submucosal connective tissue have been described in the larynx, mainly as case reports or small series including synovial sarcoma [54,55,56], well-differentiated and dedifferentiated liposarcoma [57,58,59], alveolar soft part sarcoma [60], low-grade fibro-myxoid sarcoma [61], embryonal rhabdomyosarcoma [62,63,64,65,66], Kaposi sarcoma [67], and undifferentiated sarcoma [68,69,70] (Figure 2 and Figure 3).

Synovial sarcoma represents an aggressive soft tissue tumor classically arising from mesenchymal tissue. Primary synovial sarcoma of the H&N is rare (<5% of all synovial sarcomas), and those originating from the larynx are even rarer [71]. The first case of laryngeal synovial sarcoma was described by Gatti and Miller in 1975 [72,73,74]. The diagnostic of this tumor represents a challenge, and the final diagnosis is made on the basis of tumor morphology (monophasic or biphasic according to the proportion of spindle and epithelial cells), immunohistochemistry, and molecular studies. Wide local resection is recommended as a treatment option with adjuvant RT, with or without CT, depending on final histological results [71].

Kaposi´s sarcoma is an extremely rare HIV-associated neoplasm. The largest series across the literature corresponds to Mochloulis et al., including 17 patients with laryngeal Kaposi’s sarcoma. In this series, the supraglottic location was the most common, and the main treatment strategy was conservative; more specifically, five patients were treated with low-dose radiotherapy and ten were treated with systemic chemotherapy for disseminated Kaposi’s sarcoma. According to the authors, laryngeal Kaposi’s sarcoma did not contribute to patient mortality [74]. More recently, treatment strategies using intralesional bevacizumab have also been reported as a possible treatment for localized mucosal lesions [75].

### 3.6. Bone/Cartilage Sarcomas

Demographically, these tumors usually affect patients between the sixth and seventh decades of life with a male predominance of 3–4:1 [76,77]. Chondrosarcoma is the most common sarcoma subtype [78], and the cricoid cartilage (70–75%), followed by the thyroid cartilage (20%), are the most commonly affected laryngeal subsites [54]. The majority of them are low-grade tumors with uncommon local or distant spread. Nowadays, surgery is considered the treatment of choice and, according to the disease extension, cricoarytenoid joints mobility, and histological grade, the surgical approach can range from an endoscopic approach to an open partial or total laryngectomy [54]. In one of the largest case series published by Thompson et al. including 111 laryngeal chondrosarcomas, 73 patients were treated with a partial laryngectomy while the remaining patients underwent TL; recurrence was reported in 20 patients (18% of the sample), 10 of whom underwent salvage TL [76].

Osteosarcoma of the larynx represents an extremely rare and highly malignant neoplasm [78,79], with a tendency to hematogenous dissemination and early metastasizes. Histologically, it also represents a challenge for the most experienced pathologist due to its similarities to other sarcomas. The definitive diagnosis of osteosarcoma depends on the identification of osteoid production by malignant cells in the biopsy sample. Wide local resection (TL) with clear margins remains the treatment of choice for laryngeal osteosarcoma due to the need to achieve local control and improve long-term survival [80].

Radiological evaluation can provide more information; in the case of SCC or sarcomatoid carcinoma, there is an association with the mucosal surface, whereas true sarcomas are usually centered within the soft tissue or cartilaginous structures [76]. Other radiological considerations can be the presence of calcification associated with osteoid or cartilage matrix or the presence of fat [76].

During the histological evaluation, immunostaining is crucial, and looking for cytokeratin, p63 and/or p40 could be helpful [81], as sarcomatoid carcinoma, the most common differential diagnosis, may lose expression of these markers [81].

### 3.7. Lymphoepithelial Carcinoma

Lymphoepithelial carcinoma (LC) usually arises in the lymphoid tissue of the pharynx within the Waldeyer´s ring. The presence of LC in the larynx is extremely rare. It has been hypothesized that the benign lymphoid component is geographical to the site rather than a reactive lymphocytic influx responding to a carcinoma [79]. This would explain the unusual presentation of such tumors in locations like the trachea or the larynx, which lack consistent organized lymphoid tissue.

According to the evidence available, this tumor more commonly affects male patients between 40 and 82 years. Smoke or alcohol intake has not been related with the occurrence of this kind of tumors [82,83]. The correlation between this tumor and the Epstein Barr Virus (EBV) infection is still controversial [83,84,85,86,87]. The majority differential diagnosis of lymphoepitheliomas includes non-Hodgkin’s lymphoma, rhabdomyosarcoma, and squamous cell carcinoma [82].

### 3.8. Malignant Melanoma

Regarding malignant melanoma of the larynx (MML), it is necessary to differentiate a primary mucosal malignant melanoma from a metastasis of a cutaneous malignant melanoma. In the first case, primary MML of the H&N is an uncommon disease. This entity probably derives from scattered melanocytes in the upper aero-digestive tract mucosa, which may be included within the basal layer of the epithelium of the nasal cavity, oral cavity, oropharynx, and esophagus. However, melanocytes are rarely detected within the larynx, and this could explain the low incidence of primary melanoma in the larynx [88,89,90]. By contrast, at least 0.6% of patients affected by cutaneous melanoma suffer from a metastasize to the mucosa of the upper aerodigestive tract, and of those metastatic sites, 12% are laryngeal [91].

The differential diagnosis of primary laryngeal melanoma usually includes SCC, NET, non-Hodgkin’s malignant lymphoma, extramedullary plasmacytoma, paraganglioma, and sarcomas (malignant fibrous histiocytoma, fibrosarcoma, and malignant Schwannoma) [92]. Usually, the primary treatment for MML is radical surgery with or without adjuvant radiotherapy or chemotherapy. However, endoscopic resection has been described [54]. Although radical surgery represents the best option for local control, local recurrence occurs frequently [91]. Given the small proportion of cases, optimal management is not well established, and despite controversies, it could be useful to excise isolated recurrence and metastasis, particularly if they cause airway symptoms [93].

### 3.9. Primary Lymphoma of the Larynx

Lymphoproliferative diseases like extramedullary plasmacytoma or non-Hodgkin’s lymphoma (NHL) can affect the larynx. NHL usually arises in the supraglottic and subglottic structures where laryngeal lymphoid tissues predominate [94]. These tumors are usually macroscopically smooth or polypoid [95,96,97]. The majority of reported primary lymphomas of the larynx are NHL with a B cell phenotype [98].

MALT lymphoma needs to be considered in the differential diagnosis of a patient with this presentation [99]. In the past, the standard treatment for primary lymphoproliferative tumors of the larynx was RT [99,100]. However, in the recent years, given the increasing evidence of the efficacy of systemic therapies and immunologic targeting antibodies, the tendency is moving forward in this direction.

### 3.10. Perspectives in Non-Squamous Cell Carcinomas

Regarding treatment, as we detailed above, laryngeal non-SCCs represent a significant challenge to clinicians because no definitive guideline exists. However, recent studies like the published by Landelli et al. shed some light. In this study, the authors found a significant difference among the various laryngeal subsites involved by SCC and non-SCC. The supraglottic and subglottic regions are strongly associated with non-SCC compared to SCC [17]. Furthermore, the authors did not find differences regarding overall survival or disease-specific survival between SCC and non-SCC [17]. By contrast, Chen et al. found a significant difference in OS between non-SCC and SCC with longer OS in the latter, maybe due to the differences in the proportion of cases included by etiology with more neuroendocrine carcinomas [39]. The heterogeneity in the indexed literature makes it mandatory to improve data collection in order to generate enough evidence for guidelines development.

## 4. Conclusions

To optimize overall treatment outcomes, a multidisciplinary, patient-centered approach to the management of non-SCC of the larynx must be adopted universally. National or international registry on non-SCC laryngeal cancer can be useful to improve understanding about the behavior of these kind of tumors.

## Figures and Tables

**Table 1:** Histological non-squamous cell carcinomas of the larynx.

Main Histology Group	Subtypes or Most Common Disease
**Neuroendocrine Tumors** (Epithelial and Neural)	- Typical carcinoid - Atypical carcinoid - Undifferentiated carcinoma WHO Classification (2017) - Well differentiated - Moderately differentiated - Poorly differentiated Small cell type Large cell type
**Spindle-Cell Carcinoma**	
**Minor Salivary Gland Tumors**	- Adenosquamous carcinoma - Mucoepidermoid carcinoma - Adenoid cystic carcinoma - Adenocarcinoma
**Soft Tissue Sarcomas**	- Synovial sarcoma - Well-differentiated and dedifferentiated liposarcoma - Alveolar soft part sarcoma - Low-grade fibro-myxoid sarcoma - Embryonal rhabdomyosarcoma - Kaposi sarcoma - Undifferentiated sarcoma
**Bone/Cartilage Sarcomas**	- Low-grade - High-grade
**Lymphoepithelial Carcinoma**	
**Malignant Melanoma**	- Primary mucosal malignant melanoma - Metastatic melanoma
**Primary Lymphoma of the larynx**	- Extramedullary plasmacytoma - Non-Hodgkin’s lymphoma

## Data Availability

New data were not generated in this review.
